# On the Anæsthetic Action of Apomorphine

**Published:** 1885-09-20

**Authors:** 


**On the Anæsthetic Action of Apomorphine.**—Ludwig and Burgmiester have experimentally demonstrated that the anæsthetic action of apomorphine equals, as far as the eye is concerned, that of cocaine (Centralblatt f. d. ges. Therapie, May, 1885).

After the instillation of a two per cent. solution of crystallized muriate of apomorphine (Merck's preparation) in a quantity of 6 to 18 drops, so complete an anæsthesia of the cornea and conjunctiva resulted within ten minutes that irritations of every kind provoked neither response nor any reflex activity. The animals experimented upon were cats, which are well known to react quicker on irritation of cornea or conjunctiva than rabbits.

The anæsthesia lasted usually five to ten minutes. In one case only the animal retained impaired sensibility for one hour.

Applied on man, apomorphine produces a passing, though sufficiently marked injection and pain of the conjunctival sac. Mydriasis alway attends the exhibition of this instillation.

Salivation and vomiting were so invariably noted in animals that these untoward effects were regarded as constant attendants of the application of apomorphine.

To summarize the results obtained on man, the drug was found :

1. To render the cornea and conjunctiva anæesthetic within ten minutes after the instillation of 6 to 12 drops of two per cent. solution.

2. To be painful and irritant.

3. To produce moderate mydriasis and marked nausea.

4. To cause xerosis (*i. e.*, diminution of the glandular secretion.

If it be possible to obviate these untoward effects of apomorphine, the drug would be likely to receive a direct therapeutic consideration not unlike cocaine.—*Therapeutical Gazette.*

